# *In vitro *culture of embryonic mouse intestinal epithelium: cell differentiation and introduction of reporter genes

**DOI:** 10.1186/1471-213X-6-24

**Published:** 2006-05-25

**Authors:** Jonathan M Quinlan, Wei-Yuan Yu, Mark A Hornsey, David Tosh, Jonathan MW Slack

**Affiliations:** 1 Centre for Regenerative Medicine, Department of Biology & Biochemistry, University of Bath, Bath BA2 7AY, UK; 2 Department of Craniofacial Development, King's College London, Floor 28, Guy's Hospital, London Bridge, London SE1 9RT, UK

## Abstract

**Background:**

Study of the normal development of the intestinal epithelium has been hampered by a lack of suitable model systems, in particular ones that enable the introduction of exogenous genes. Production of such a system would advance our understanding of normal epithelial development and help to shed light on the pathogenesis of intestinal neoplasia. The criteria for a reliable culture system include the ability to perform real time observations and manipulations *in vitro*, the preparation of wholemounts for immunostaining and the potential for introducing genes.

**Results:**

The new culture system involves growing mouse embryo intestinal explants on fibronectin-coated coverslips in basal Eagle's medium+20% fetal bovine serum. Initially the cultures maintain expression of the intestinal transcription factor Cdx2 together with columnar epithelial (cytokeratin 8) and mesenchymal (smooth muscle actin) markers. Over a few days of culture, differentiation markers appear characteristic of absorptive epithelium (sucrase-isomaltase), goblet cells (Periodic Acid Schiff positive), enteroendocrine cells (chromogranin A) and Paneth cells (lysozyme).

Three different approaches were tested to express genes in the developing cultures: transfection, electroporation and adenoviral infection. All could introduce genes into the mesenchyme, but only to a small extent into the epithelium.

However the efficiency of adenovirus infection can be greatly improved by a limited enzyme digestion, which makes accessible the lateral faces of cells bearing the Coxsackie and Adenovirus Receptor. This enables reliable delivery of genes into epithelial cells.

**Conclusion:**

We describe a new *in vitro *culture system for the small intestine of the mouse embryo that recapitulates its normal development. The system both provides a model for studying normal development of the intestinal epithelium and also allows for the manipulation of gene expression. The explants can be cultured for up to two weeks, they form the full repertoire of intestinal epithelial cell types (enterocytes, goblet cells, Paneth cells and enteroendocrine cells) and the method for gene introduction into the epithelium is efficient and reliable.

## Background

The gastrointestinal tract of vertebrates is lined by different epithelia that develop from the embryonic endoderm under the influences of developmental signals from the associated splanchnic mesoderm [1]. In the developing rodent intestine, the epithelium is initially composed of a pseudostratified layer which converts into a single-layered epithelium at around embryonic day 13.5–14.5 (E13.5–14.5d) [2]. The other cell layers of the gut are derived from the mesoderm. The epithelium, together with the underlying connective tissue (the lamina propria) and a thin muscle layer (the muscularis mucosa), is referred to as the mucosa.

The adult intestinal epithelium is organized into villi, which project into the gut lumen. At the base of the villi are the crypts of Lieberkuhn which contain the stem cell compartment [3-5]. There are three differentiated cell types located along the villus. These are the enterocytes, goblet cells and enteroendocrine cells. The enterocytes are absorptive and secrete enzymes such as sucrase-isomaltase [6]. Goblet cells secrete mucus thereby providing a protective lining for the intestinal cells against the proteolytic action of the digestive enzymes. The enteroendocrine cells secrete hormones including secretin and glucagon-like peptide-1 [7,8]. In addition, Paneth cells are found at the bottom of the crypts. These secrete antimicrobial agents such as lysozyme, cryptidins and defensins [9]. All four epithelial cell types are believed to differentiate from common pluripotent stem cells located in the crypt compartment of the intestine [10,11].

Study of the normal development of the intestinal epithelium has been hampered by lack of suitable model systems [12], in particular ones that enable the introduction of exogenous genes. Production of such a system would advance our understanding of normal epithelial development and help to shed light on the pathogenesis of intestinal neoplasia. Several attempts have been made to produce culture systems that mimic normal intestinal development. The main problem is that once intestinal cells are removed from the basement membrane and underlying stroma, apoptosis is initiated within a few hours [13,14]. Explants of embryonic gut will develop successfully when transplanted under the kidney capsule of syngeneic or immunocompromised hosts [15] or the coelomic cavity [16]. However, with such *in vivo *cultures the tissue is then inaccessible to study. A recently developed *in vitro *system involves culture of embryonic intestinal explants by attachment to filters (catenary cultures) [17-19]. Some morphogenesis of the tissue was observed but the authors did not look at the full complement of epithelial cell types. Abud and colleagues [18] reported some success introducing genes by electroporation but we have not been able to replicate these results (see below).

We sought to develop an *in vitro *organ culture system in which cultures were easily established and the culture conditions supported the differentiation of the various intestinal cell lineages. The criteria for a reliable culture system include: the ability to perform real time observations and manipulations *in vitro*, the preparation of wholemounts for immunostaining and the potential for introducing genes. We have previously described the development of *in vitro *systems for mouse embryonic pancreas and oesophagus based on culture of tissue explants on fibronectin-coated glass coverslips [20-23]. We have now extended this culture system to intestine and furthermore developed a new procedure for gene introduction involving enzyme treatment and adenovirus infection. This culture system meets all of the criteria. The explants can be cultured for up to two weeks, they form the full repertoire of intestinal epithelial cell types (enterocytes, goblet cells, Paneth cells and enteroendocrine cells) and the method for gene introduction into the epithelium is efficient and reliable.

## Results

### Development of intestinal embryonic tissue *in vitro*

**Figure 1:**
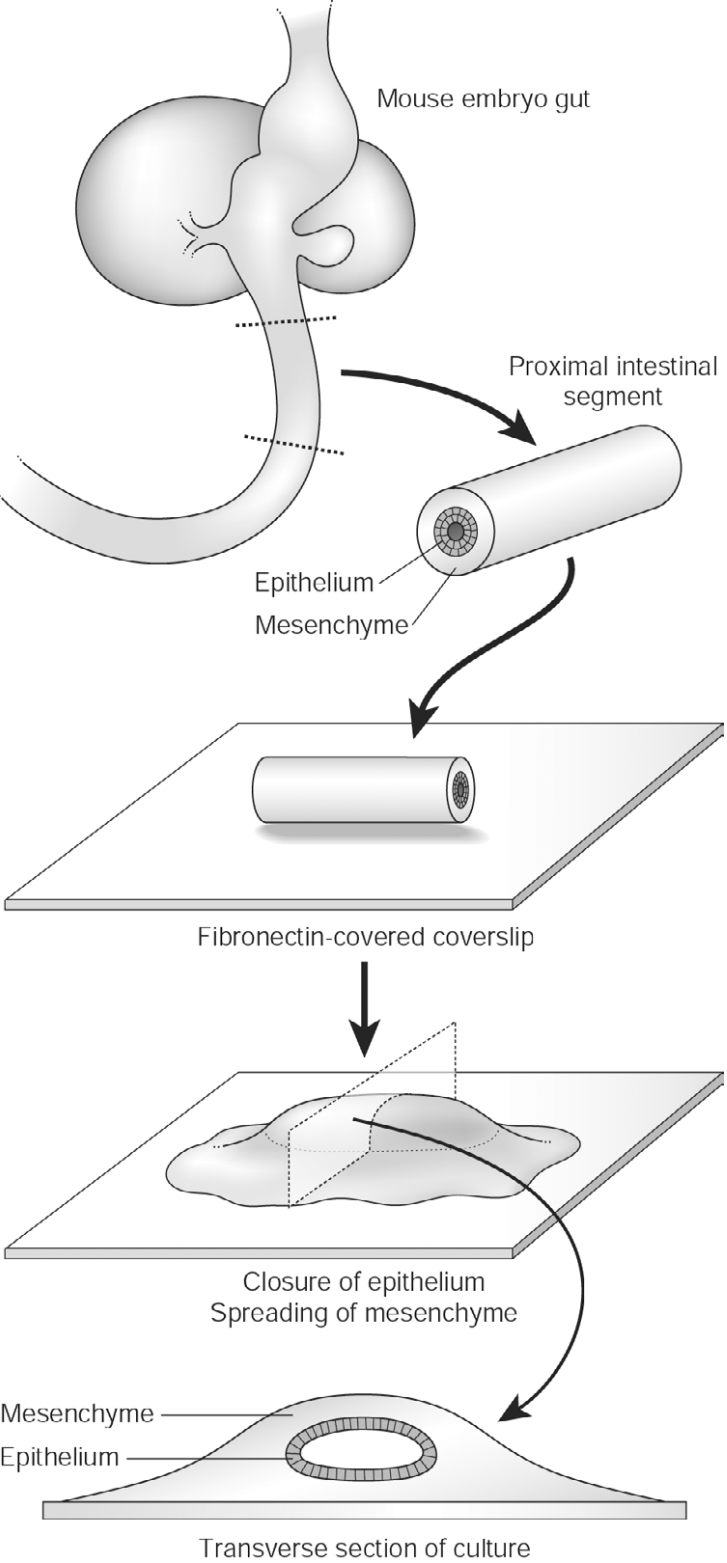
The establishment of an intestinal explant culture and its internal structure.

**Figure 2:**
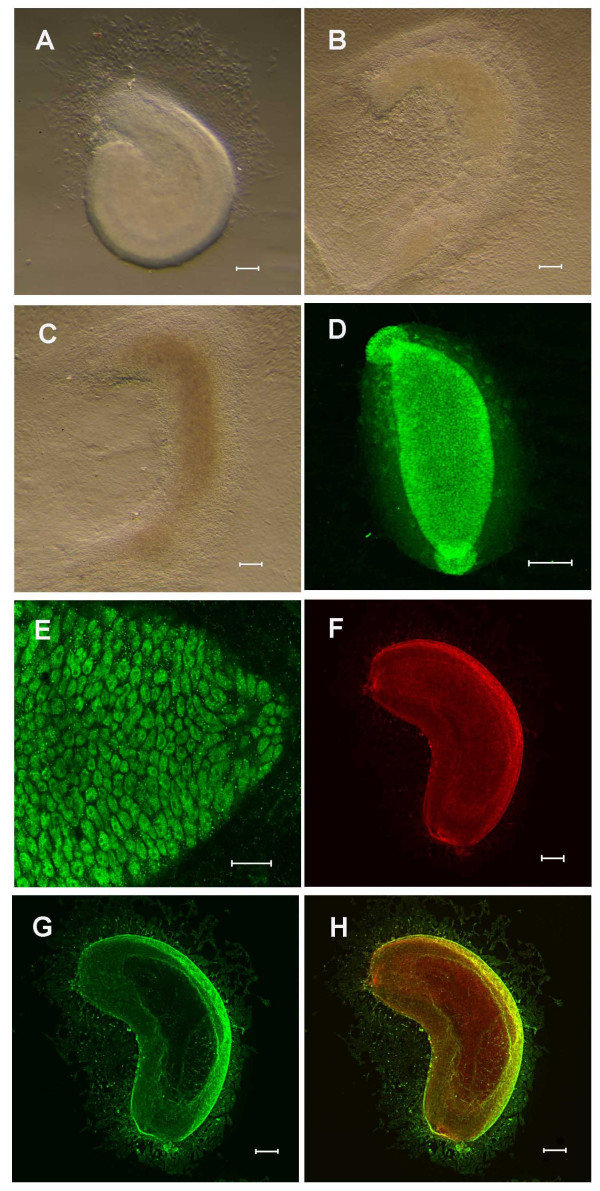
**Development of the murine embryonic intestine *in vitro***. A-C) Phase contrast images of small intestine after 1 (A), 7 (B) and 11 (C) days of culture. An epithelial tube persists in the centre, with the surrounding mesenchyme spreading out to form a monolayer. D-E) Cdx2 expression in 1 day cultures, wholemount (D) and confocal section through epithelium (E). F-H) Epithelium and mesenchyme form concentric layers. The columnar epithelium is visualised by immunostaining for cytokeratin 8 (F) and the mesenchyme by staining for smooth muscle actin (G). Scale bars = 100 μm A-D, F-H; 20 μm E.

E13.5d intestinal explants were isolated and grown in culture for up to 11 days (Fig 1). Intestinal tissue from younger embryos (E11.5d) was also used successfully (results not shown). We investigated the development *in vitro*, initially focusing on morphology and differentiation. The explants adhere to the fibronectin substrate within a few hours and gradually flatten out over the first 1–2 days. The cut ends of the epithelium close and the mesenchymal cells spread rapidly out of the explant to form a monolayer of cells on the substrate, with several cell layers surrounding the epithelial tube in the centre. Over the next few days, the epithelium extends and the mesenchyme continues to expand, increasing the area of the culture (Fig 2A–C). The flattening of the whole culture with time is accompanied by collapse of the lumen although this remains as a virtual space, i.e. with opposing epithelial layers apposed. Initially the explants did not exhibit any contractile activity but after 2–3 days in culture, random peristaltic movements were apparent. These are slow contractions lasting a couple of seconds and occurring once or twice a minute.

### Expression of intestinal markers in the epithelium

**Figure 3:**
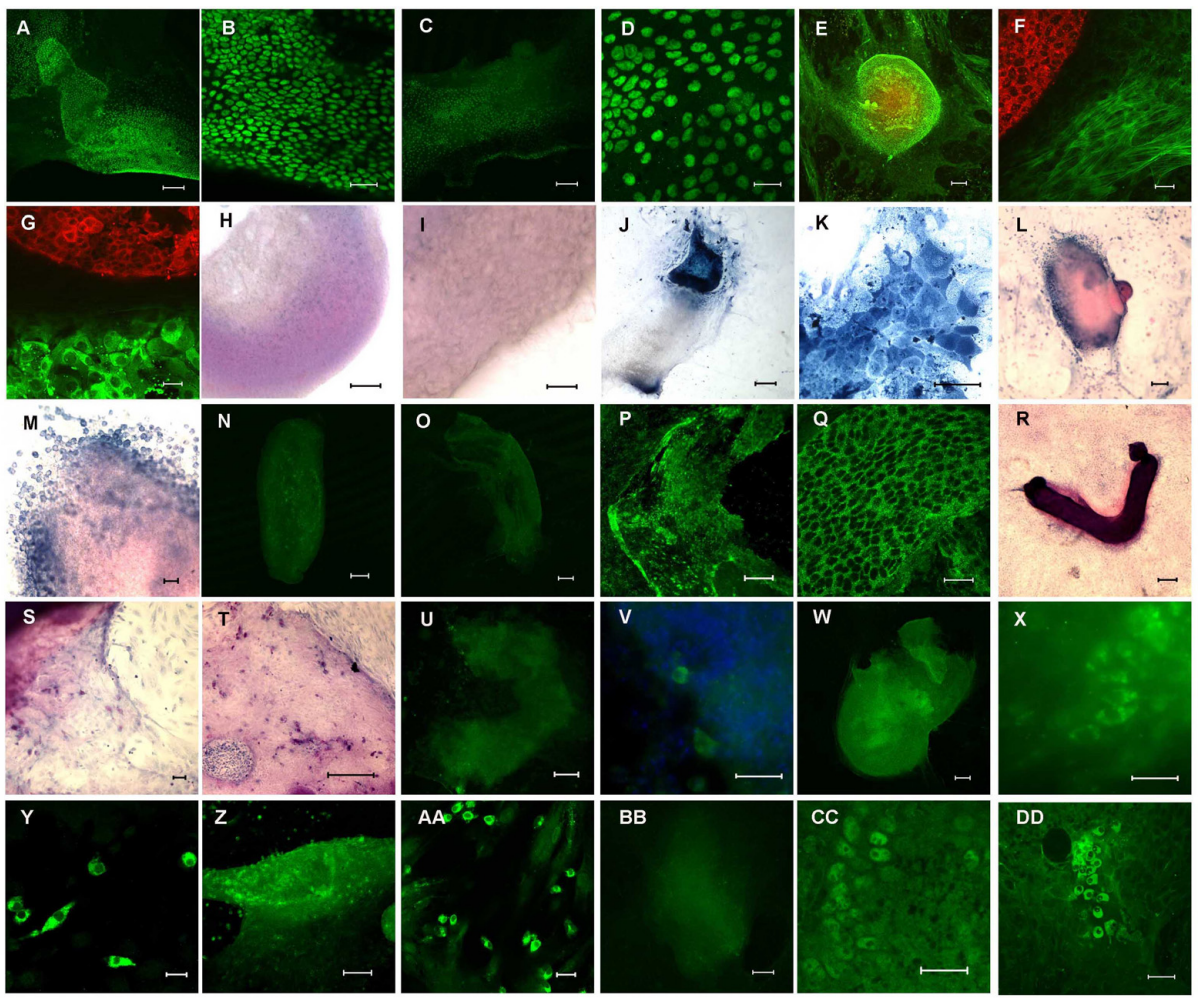
**Expression of intestinal markers in culture**. A-D) Continued Cdx2 expression after 7 (A, B) and 11 (C, D) days of culture. E-G) Concentric expression of the columnar epithelial marker cytokeratin 8 (red) and mesenchymal smooth muscle actin (green) persists on day 7 (E, F) and 11 (G) of culture. H-DD) Expression of differentiated epithelial markers. H-M) alkaline phosphatase, visualised by histochemistry, is absent after 1 day of culture (H, I) and is expressed in enterocytes after 7 (J, K) and 11 days (L, M). N-Q) Sucrase-isomaltase, visualised by immunostaining, is absent at 1 (N) and 7 days (O), thereafter demonstrated in enterocytes at day 11 of culture (P, Q). R-T) Goblet cells visualised by PAS staining are seen at 7 days (R, S) and persist for the duration of culture (T). U-X) Chromogranin A, visualised by immunostaining, is expressed in enteroendocrine cells at 7 (U, V) and 11 (W, X) days. Y-AA) The antimicrobial agent lysozyme is demonstrated by immunostaining in Paneth cells at 7 (Y) and 11 (Z, AA) days. BB-DD) HNF-4 α expression, visualised by immunostaining, shows a similar pattern (BB, CC 7 days; DD 11 days of culture). Scale bars = 100 μm A, C, E, H, J, L, N-P, R, T, U, W, Z, BB; 20 μm B, D, F, G, I, K, M, Q, S, V, X, Y, AA, CC, DD.

We initially anlaysed the expression of the intestinal transcription factor Cdx2. The *cdx2 *gene encodes a homeodomain transcription factor whose expression normally distinguishes between the upper and lower regions of the alimentary canal epithelia [24,25]. Cdx2 is expressed *in vivo *by E9.5d [24]. Twenty-four hours after isolation, the epithelium of the E13.5d intestinal explants still expressed Cdx2 (Fig 2D,E) and this expression persisted for the duration of the culture period (Fig 3A–D).

We proceeded to examine the expression of epithelial and mesenchymal markers in the cultures. Simple columnar epithelia express cytokeratin 8 (K8), a type II acidic protein that normally heterodimerises with another intermediate filament, the type I cytokeratin 18 (K18) to form the keratin intermediate filament structure [26]. Fig 2F–H shows a specimen cultured for one day then stained for K8 to show the columnar epithelium and smooth muscle actin to show the mesenchyme. This concentric arrangement of epithelium and mesenchyme persisted for the duration of the culture period (Fig 3E–G).

As previous studies have only described limited characterisation of differentiation, we studied the formation of all four differentiated epithelial cell types in the culture system using various markers (Fig 3H–DD). The enterocyte marker alkaline phosphatase was detected by day 7 in culture by histochemical staining and sucrase-isomaltase by day 11. PAS-positive goblet cells were demonstrated at day 7. Chromogranin A, produced by enteroendocrine cells, and lysozyme-positive Paneth cells were both detected by day 7. The transcription factor hepatocyte nuclear factor 4α (HNF-4α), a member of the steroid hormone receptor superfamily important in regulating intestinal gene expression, was also seen by day 7 in culture. The appearance of these markers is consistent with development *in vivo *[27-29] and, once present, all the markers persisted for the duration of the culture period.

### Ectopic gene expression in intestinal explants

**Figure 4:**
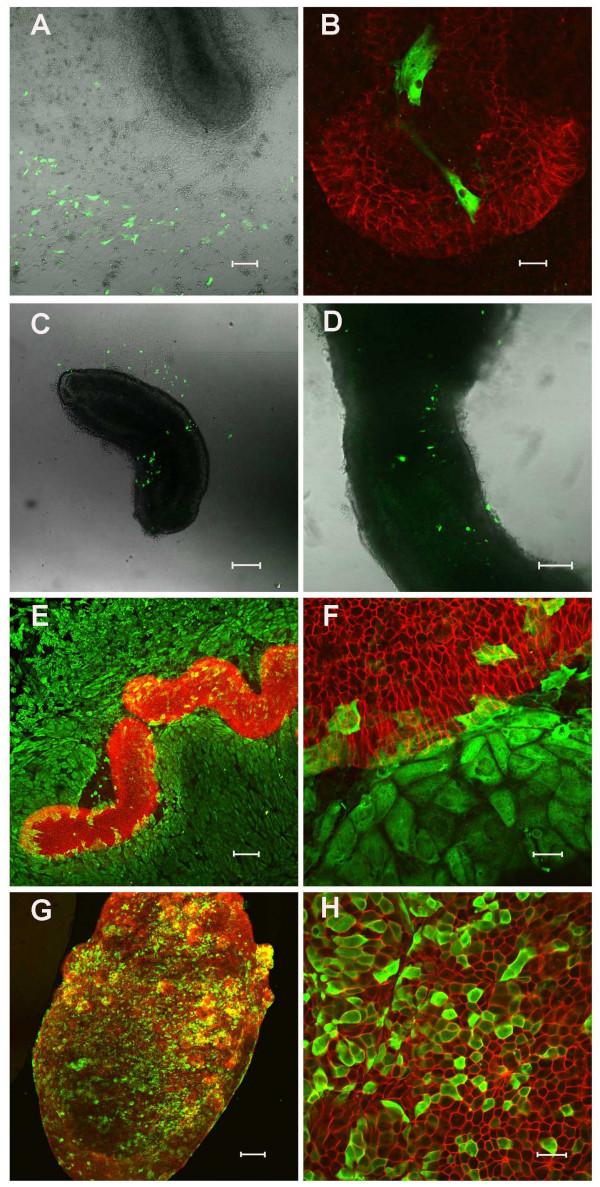
**Gene delivery to embryonic intestine**. (A) and (B) DsRed expression 24 hours after GeneJuice transfection (DsRed is here coloured green to permit direct comparison with the images in the rest of the figure). There is no expression of DsRed in the epithelium (E-cadherin, red). (C) and (D) Electroporation of *pcDNA3nucGFP2 *into the intestine. GFP is visualised predominantly in the mesenchyme after 24 hours culture. (E) and (F) Infection of intestine explants with *Ad-CMV-LacZ *adenoviral vector. E-cadherin is shown in red and β-galactosidase in green 48 hours after infection. (G) and (H) Pretreatment of intestinal explants with dispase substantially enhances adenoviral-mediated gene delivery to the epithelium. E-cadherin is shown in red and β-galactosidase in green. Representative cultures are shown for each regime. Scale bars = 100 μm A, C-E, G; 20 μm B, F, H.

We were especially interested in the potential of the intestinal culture system for ectopic gene expression. We initially tried a commercially available transfection reagent, GeneJuice, to introduce *DsRed *into the intestinal epithelium. Mesenchymal but not epithelial expression of DsRed could be detected under epifluorescence following 24 hours of culture (Fig 4A,B). Since there was no epithelial expression we decided to examine alternative methods. Electroporation has been successfully developed for manipulating gene expression in chick [30,31] and there have been reports of electroporation of mouse tissues [18,32]. We tested a wide variety of electroporation conditions using the *pcDNA3nucGFP2 *plasmid. The optimum condition was 40 V, 3 pulses of 50 ms, pulse interval 500 ms, but even with these settings only 0.9 ± 0.3% epithelial cells in the centre of the cultures took up the plasmid and expressed GFP (Fig 4C,D). A number of GFP-positive cells were also found in the mesenchyme.

Since epithelial expression was poor both with transfection and electroporation, we set out to determine whether active genes could be introduced into the epithelium of the cultures using adenoviral vectors. We infected intestinal epithelium with a first generation adenovirus expressing *LacZ *as described in the Methods. The intestinal explants were opened to ensure good access to the epithelium then exposed to high titre virus for 2 hours. We observed abundant expression of β-galactosidase in the mesenchyme (Fig 4E,F) but only in 2.3 ± 1.3% cells of the epithelium (Table 2). Adenovirus infection requires the interaction of a viral coat protein with the Coxsackie and Adenovirus Receptor (CAR) in the target cell. In polarised epithelial cells such as those of the intestine, CAR is found on the lateral membranes associated with the tight junctions [37]. Because of this we reasoned that a light enzyme digestion might succeed in opening up the tight junctions, so allowing access of the virus to CAR, while not degrading the tissue so much that the epithelium broke down. A number of trials were made with enzymes including trypsin and collagenase, but the best results were obtained using dispase. As before, the intestinal segments were opened, and then treated at various times and concentrations, followed by application of the virus. The optimum regime was a 1–2 minute treatment with dispase at 50 caseinolytic units/ml. This enhanced β-galactosidase expression in the epithelium, verified by the co-expression with E-cadherin (Fig 4G,H).

**Table 1:** Antibodies

**Experiment**	**Primary**	**Supplier**	**Secondary**	**Supplier**
AdLacZ	mouse monoclonal anti-E-cadherin 1/50	BD Transduction Laboratories	horse anti-mouse Texas Red conjugated IgG 1/100	Vector
	rabbit polyclonal anti-β-galactosidase 1/300	ICN	goat anti-rabbit fluorescein isothiocyanate (FITC) conjugated IgG 1/100	Vector
GeneJuice	mouse monoclonal anti-E-cadherin 1/50	BD Transduction Laboratories	horse anti-mouse FITC conjugated IgG 1/100	Vector
	rabbit polyclonal anti-DsRed 1/3000	Clontech	Swine anti-rabbit tetramethylrhodamine isomer R (TRITC) conjugated IgG 1/100	DAKO
Small Intestine characterisation	Rat monoclonal anti-E-cadherin 1/100	Zymed	goat anti-rat Texas Red conjugated IgG 1/100	Vector
	rat monoclonal anti-Troma-1 (cytokeratin 8) 1/50	DSHB, University of Iowa	goat anti-rat Texas Red conjugated IgG 1/100	Vector
	mouse monoclonal anti-α smooth muscle actin 1/100	Sigma	horse anti-mouse FITC conjugated IgG 1/100	Vector
	mouse monoclonal anti-sucrase-isomaltase 1/200	Gift (Dr Hans-Peter Hauri)	horse anti-mouse FITC conjugated IgG 1/100	Vector
	mouse monoclonal anti-cdx2 1/100	BioGenex	horse anti-mouse FITC conjugated IgG 1/100	Vector
	rabbit polyclonal anti-lysozyme 1/100	DAKO	goat anti-rabbit FITC conjugated IgG 1/100	Vector
	rabbit polyclonal anti-chromogranin A 1/100	DAKO	goat anti-rabbit FITC conjugated IgG 1/100	Vector
	goat polyclonal anti-HNF-4α 1/100	Santa Cruz	rabbit anti-goat FITC conjugated IgG 1/100	Vector

**Table 2:** Percentage of labeled epithelium following transfection, electroporation or adenoviral infection. The percentage of epithelial cells labeled represents the average counts from 100 cells over five fields from three different buds.

	**% explants with any expression**	**% explants with epithelial expression**	**% epithelial cells**
**GeneJuice**	75 (3/4)	0 (0/4)	0
**Electroporation**	73.8 (31/42)	11.9 (5/42)	0.9 ± 0.3
**AdLacZ - Dispase**	100 (24/24)	25 (6/24)	2.3 ± 1.3
**AdLacZ + Dispase**	100 (25/25)	68 (17/25)	29.1 ± 1.1***

*** p < 0.001. One-way ANOVA (non-parametric). SEM given for % epithelial cells labeled.

Table 2 shows the percentage of labeled epithelium following transfection, electroporation or adenoviral infection, showing quantitatively that infection with adenovirus following dispase treatment is the most efficient method (p < 0.001).

## Discussion

Many previous *in vitro *culture systems have been utilized to investigate intestinal development. Such systems include embryonic gut transplanted under the kidney capsule of syngeneic or immunocompromised hosts [15], intestinal explants attached to filters [17-19] or small slices or biopsies of fetal or postnatal intestinal tissue cultured *in vitro *for 3 days [38-40]. However, these models suffer from a number of different problems. Tissue cultured using these techniques are not accessible to experimental manipulation and the medium surrounding the explants cannot be manipulated. In some of these systems [38-40], differentiation of the cultures is poor due to reduced viability and only limited differentiation has been documented in other organ systems. In the present system, we provide evidence for the differentiation of the embryonic intestine based on the expression of markers of epithelial cell types. We therefore conclude that the differentiation program is normal.

Although we tried a number of techniques for overexpression including transfection, electroporation and adenoviral infection, none of them produced significant epithelial expression. This was somewhat surprising given the previous published protocols [18]. We therefore sought to improve on the adenoviral system. We believe that the dispase digestion method works because it slightly separates the cells and makes accessible the lateral faces which carry the adenovirus receptor. This dispase/adenovirus method provides the first reliable technique for introducing exogenous genes into the intestinal epithelium. Because the action of many genes can be specifically antagonised by dominant negative constructs or siRNA, gene introduction can also enable inhibition of the action of specific endogenous genes. This new method will provide an additional resource to study changes in intestinal gene expression and differentiation, and will enable the study of intestinal development by both gene overexpression and specific ablation.

## Conclusion

The current system offers a number of advantages to those previously described. It (i) is simple and depends largely on the presence of a suitable substrate and a simple medium (ii) maintains the gut tube with intact mesenchymal and epithelial layers (iii) permits differentiation of all of the normal intestinal cell types on a normal time scale (iv) remains accessible to observation and manipulation during the culture period (v) is suitable for wholemount immunostaining, providing three-dimensional visualisation of anatomical features and (vi) is suitable for ectopic gene expression.

## Methods

### Mice and isolation of embryos

Animal husbandry and embryo isolation were carried out in accordance with UK Home Office regulations. Stage-specific embryos were isolated from timed matings with CD1 mice, based on the observation of a copulatory plug representing E0.5d. Pregnant animals were killed by cervical dislocation and the uteri dissected free into ice-cold sterile phosphate buffered saline A (PBSA). E13.5d embryos were removed from the decidua, transferred to ice-cold Minimum Essential Medium (MEM) with Hanks' salts (Sigma), 10% fetal bovine serum (FBS, Invitrogen), 2 mM L-glutamine (Sigma) and 20 μg/ml gentamicin (Invitrogen) and the gut (from the pharynx to the intestine) was dissected free. An intestinal segment was then removed as shown in Fig 1.

### Isolation and culture of embryonic intestine

E13.5d intestines were cultured on coverslips subbed with 3-aminopropyltriethoxysilane (APTES, Sigma) and coated with bovine plasma fibronectin (Invitrogen) at 50 μg/ml in sterile water [23]. Initially, a cloning ring was placed over the fibronectin-coated area in order to ensure that the explant stayed on the substrate. Basal Medium Eagle (BME) with Earle's salts (Sigma), 20% FBS, 2 mM L-glutamine and 20 μg/ml gentamicin was pipetted dropwise into the cloning ring. Twenty four hours later the cloning ring was removed, the medium decanted and fresh medium added. The cultures were grown at 37°C, 95% air/5% CO_2 _in a humidified incubator for up to 11 days. The medium was changed every 2 days.

### Histology and immunohistochemistry

We performed immunohistochemistry on wholemounts as described previously[33,34]. Briefly, intestinal cultures (1–11 days) were fixed for 5 minutes in acetone/methanol (1:1 ratio) at -20°C for immunostaining of cytoskeleton proteins or 30 minutes in MEMFA pH7.4 (3.8% formaldehyde, 0.15 M MOPS, 2 mM EGTA, 1 mM MgSO_4_) at room temperature for immunostaining of membrane, cytosolic and nuclear proteins. After fixation, cultures were washed three times in PBSA and stored in PBSA at 4°C. Cultures were permeabilised by adding 1% Triton X-100 (Sigma) in PBSA for 30 minutes prior to immunostaining. Antigen retrieval was performed using citrate buffer pH6 (Lab Vision Corporation) for 1 hour at 37°C. The cultures were washed three times in PBSA. Non-specific binding sites were blocked for at least 1 hour in 2% Blocking Reagent (Roche). Primary antibodies were applied overnight at 4°C and the following day the samples were washed three times in PBSA. The fluorescent secondary antibody was applied for 3 hours in the dark at room temperature, and the coverslips were rewashed and then mounted in Gel/Mount (Biomeda corp).

### Microscopy and photography

During the culture period, the live cultures were observed using a Leica DMIRB inverted microscope and the immunostained or histochemically stained wholemounts were visualised under a Leica DMRB compound microscope. The images were taken using a color SPOT RT camera (Diagnostic Instruments) operated with Advanced Spot RT 3.0 software. A Zeiss LSM510 confocal microscope was used to take high resolution images of optical sections. The images were cropped and arranged using Adobe Photoshop 7.0.

### Antisera

The antibodies were obtained and diluted as described in Table 1.

### Histochemical methods

#### Enterocytes

Vector Blue alkaline phosphatase substrate kit was used according to the manufacturer's instructions. Briefly, 2 drops of reagent 1 were added to 5 ml 100 mM Tris-HCl pH8.2, followed by 2 drops of reagent 2 and 2 drops of reagent 3. Embryonic buds were incubated for 30 minutes at room temperature in the dark, washed in PBSA for 5 minutes, rinsed in tap water before dehydration and mounting in DePex (BDH).

#### Goblet cells

Goblet cells were stained by the Periodic Acid Schiff (PAS) method. MEMFA-fixed embryonic buds were washed in running tap water for 3 minutes, then treated with 1% periodic acid (BDH) for 10 minutes at room temperature, washed for 3 minutes in water and then immersed in Schiff's reagent (Sigma) in the dark for 2 minutes. Samples were washed in running tap water for 20 minutes and then 3 minutes in distilled water. The buds were then counterstained with haematoxylin before dehydration and mounting in DePeX.

### Overexpression of reporter genes

#### Transfection protocol

E13.5d small intestine explants were isolated and cultured for 48 hours as described above. For each explant, 3 μl of GeneJuice Transfection Reagent (Novagen) was added to 100 μl of Optimem I medium (Gibco) and mixed by vortexing. The mixture was then incubated for 5 minutes at room temperature in semi-darkness. 1 μg *pcDNA3DsRed *was added to the tube and incubated for a further 15 minutes at room temperature. The culture medium was changed and, keeping the cloning ring in position, the transfection complex was added dropwise into the cloning ring. Cultures were then incubated in 95% air/5% CO_2 _at 37°C for 24 hours before MEMFA fixation and immunostaining.

### Electroporation conditions

*pcDNA3nucGFP2 *was constructed by subcloning *nucGFP2 *as a *Bam*HI/*Xba*I fragment into *pcDNA3 *(Invitrogen). 0.01% Fast Green was added to 1 μg/μl *pcDNA3nucGFP2 *plasmid DNA for easy visualization. Borosilicate standard wall (1.0 mm O.D./0.58 mm I.D.) glass capillaries (Harvard Apparatus) were pulled using a P-97 micropipette puller (Sutter Instrument Co.) with the following conditions: heat = ramp+25, pull = 20, velocity = 255, time = 150. The micropipette was back-filled with DNA solution and Fast Green, DNA injected into the lumen of the isolated intestine and electroporation performed with an Electro Square Porator (BTX, ECM830) using an electrode gap width of 4 mm. The voltage was varied from 25–100 V in a unipolar direction and the number of pulses from 3x–6x. The tissues were then examined for GFP expression by confocal microscopy after 24 hours of culture. The following settings proved optimal in terms of epithelial cells expressing GFP: 40 V, 3 pulses of 50 ms, pulse interval 500 ms.

### Recombinant adenoviral vectors

A first-generation, replication-defective, recombinant adenoviral vector was used in these experiments. *Ad-CMV-LacZ *was constructed according to a standard protocol using two plasmids, *pJM17 *and *pXCX2*, as described previously [35]. The *Ad-CMV-LacZ *adenovirus was propagated in the E1-containing human embryonic kidney (HEK) cell line 293 [36]. HEK 293 cells were maintained in Dulbecco's Modified Eagle Medium (Invitrogen) supplemented with 10% heat-inactivated fetal calf serum (Sigma). *Ad-CMV-LacZ *contains the constitutive cytomegalovirus (*CMV*) promoter driving the expression of the *Escherichia coli*-derived β-galactosidase gene (*LacZ*). To make it, the *CMV *promoter was cloned into the Klenow-blunted *Xba*I site of *pXCX2*. The resulting plasmid was cut with *Hind*III/*Bam*HI and a 3.7 kb *Hind*III/*Bam*HI fragment from *pCH110 *(Pharmacia) containing *LacZ *and an SV40 polyadenylation signal was ligated in. The resulting plasmid was cotransfected with *pJM17 *into HEK 293 cells by calcium phosphate precipitation and the cells were overlaid with agarose. Individual viral plaques were picked and plaque-purified three times. The DNA structure of the new viral vector was verified by restriction diagnostics. The virus was grown to high titre, released from the cells by rapidly freeze/thawing three times and then purified twice by caesium chloride density ultra-centrifugation. The buffer was exchanged with 10 mM Tris.HCl pH 7.5, 1 mM MgCl_2 _on a PD-10 Sephadex column (Amersham Biosciences), before the virus was passed through a sterile 0.22 μm Millex filter (Millipore) and frozen in 10 μl aliquots using dry-ice. The viral stocks were stored at -80°C. The titer of the vector was 7 × 10^9 ^infectious units/ml, which was calculated using the Adeno-X™ Rapid Titer Kit (Stratagene).

### *Ad-CMV-LacZ *infection

E13.5d small intestine was isolated as previously described and opened with a tungsten needle to expose the luminal epithelium. The segments were then incubated in dispase (activity 50 caseinolytic units/ml (BD Biosciences)) at 37°C for 1 to 2 minutes to improve viral access to the epithelium. The reaction was terminated by washing the intestine in supplemented BME and the organ buds transferred to the centre of a cloning ring. 20 μl *Ad-CMV-LacZ *was added to the cloning ring and incubated in 95% air/5% CO_2 _at 37°C for 2 hours. A further 40 μl supplemented BME was then added to the cloning ring. The culture medium was changed at 24 hours and the cloning ring removed. Cultures were maintained for up to 7 days with medium changes (2 ml) every 2 days and fixed in MEMFA.

### Immunostaining of infected explants

MEMFA-fixed intestinal cultures were treated with acetone/methanol 1:1 at -20°C for 5 minutes and immunostained as described above.

## Authors' contributions

WYY and JMQ carried out the trials of culture conditions, studies of differentiation and gene introduction. MAH prepared the recombinant adenovirus. DT and JMWS conceived of the study, and supervised its execution. The manuscript was mostly written by JMQ and JMWS, with input from all authors. All authors read and approved the final manuscript.
